# Validation of a Preformulated, Field Deployable, Recombinase Polymerase Amplification Assay for *Phytophthora* Species

**DOI:** 10.3390/plants9040466

**Published:** 2020-04-07

**Authors:** Austin G. McCoy, Timothy D. Miles, Guillaume J. Bilodeau, Patrick Woods, Cheryl Blomquist, Frank N. Martin, Martin I. Chilvers

**Affiliations:** 1 Department of Plant, Soil and Microbial Sciences, Michigan State University, East Lansing, MI 48824, USA; mccoyaus@msu.edu; 2 Ottawa Plant Laboratory, Canadian Food Inspection Agency, 3851 Fallowfield Road, Ottawa, ON K2H 8P9, Canada; Guillaume.Bilodeau@canada.ca; 3 Plant Pest Diagnostics Branch, California Department of Food and Agriculture, Sacramento, CA 95832, USA; patrick.woods@cdfa.ca.gov (P.W.); cheryl.blomquist@cdfa.ca.gov (C.B.); 4 Crop Improvement and Protection Research Unit, U.S. Department of Agriculture Agricultural Research Service, Salinas, CA 93905, USA

**Keywords:** diagnostics, isothermal amplification, oomycetes, point of care diagnostics

## Abstract

Recombinase polymerase amplification (RPA) assays are valuable molecular diagnostic tools that can detect and identify plant pathogens in the field without time-consuming DNA extractions. Historically, RPA assay reagents were commercially available as a lyophilized pellet in microfuge strip tubes, but have become available in liquid form more recently—both require the addition of primers and probes prior to use, which can be challenging to handle in a field setting. Lyophilization of primers and probes, along with RPA reagents, contained within a single tube limits the risk of contamination, eliminates the need for refrigeration, as the lyophilized reagents are stable at ambient temperatures, and simplifies field use of the assays. This study investigates the potential effect of preformulation on assay performance using a previously validated *Phytophthora* genus-specific RPA assay, lyophilized with primers and probes included with the RPA reagents. The preformulated lyophilized *Phytophthora* RPA assay was compared with a quantitative polymerase chain reaction (qPCR) assay and commercially available RPA kits using three qPCR platforms (BioRad CFX96, QuantStudio 6 and Applied Biosystems ViiA7) and one isothermal platform (Axxin T16-ISO RPA), with experiments run in four separate labs. The assay was tested for sensitivity (ranging from 500 to 0.33 pg of DNA) and specificity using purified oomycete DNA, as well as crude extracts of *Phytophthora*-infected and non-infected plants. The limit of detection (LOD) using purified DNA was 33 pg in the CFX96 and ViiA7 qPCR platforms using the preformulated kits, while the Axxin T16-ISO RPA chamber and the QuantStudio 6 platform could detect down to 3.3 pg with or without added plant extract. The LOD using a crude plant extract for the BioRad CFX96 was 330 pg, whereas the LOD for the ViiA7 system was 33 pg. These trials demonstrate the consistency and uniformity of pathogen detection with preformulated RPA kits for *Phytophthora* detection when conducted by different labs using different instruments for measuring results.

## 1. Introduction

Oomycetes within the genus *Phytophthora* constitute a large group of destructive plant pathogens. *Phytophthora* species cause root, crown, stem, foliar and fruit diseases on agriculturally and ecologically important species of plants [1,2,3,4]. These diseases can be difficult or impossible to distinguish by symptoms alone and in-lab diagnostic testing is required for accurate pathogen identification. Identification of ambiguous *Phytophthora* species has traditionally relied on techniques such as baiting, isolation onto a *Phytophthora* semi-selective medium, DNA extraction and polymerase chain reaction (PCR) or antibodies (i.e., immunostrips) to identify the genus or species present [5,6,7,8]. However, this is time consuming and some species, such as the causal agent of sudden oak death, *Phytophthora ramorum* [9], are of regulatory importance, requiring a rapid and accurate identification. Likewise, the generic antibody used in commercial immunostrips to detect *Phytophthora* species cross reacts with some *Pythium* or *Phytopythium* species, making this detection method fast but not specific [10].

**Figure 1:**
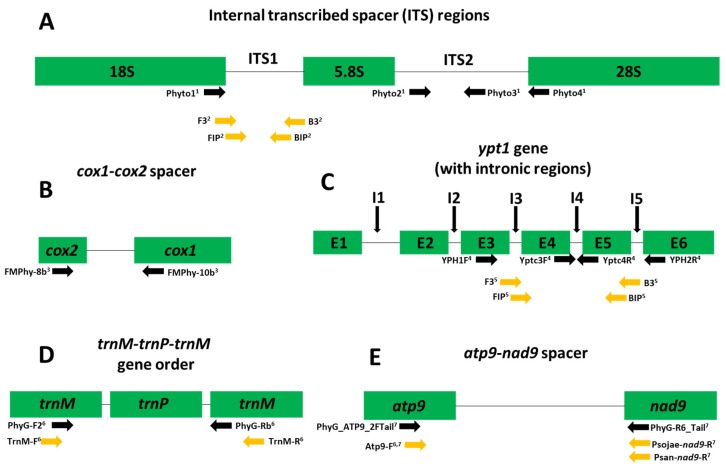
Visualization of intergenic regions used for diagnostic assays of oomycetes with particular emphasis on *Phytophthora* species. (**A**) Internal transcribed spacer region, (**B**) *cox1*-*cox2* spacer region, (**C**) *ras*-related *ypt1* gene with intronic regions, (**D**) *trnM-trnP-trnM* gene order and (**E**) *atp9*–*nad9* spacer region. Also denoted are the primers used in various reported isothermal (yellow arrows) and non-isothermal (black arrows) assays for *Phytophthora* species. ^1^ *Phytophthora ramorum*-specific primers from Garbelotto et al., 2002. Phyto2 and Phyto3 are nested primers to be used after a preliminary amplification with Phyto1 and Phyto4. ^2^ *Phytophthora ramorum*-specific primers from Tomlinson et al., 2010. ^3^ *Phytophthora* genus-specific primers from Martin et al., 2004. ^4^ *Phytophthora kernoviae*-specific primers from Schena et al., 2006. Yptc3F and Yptc4R are nested primers to be used after a preliminary amplification with YPH1F and YPH2R. ^5^ *Phytophthora infestans*-specific primers from Khan et al., 2017. ^6^ Universal primers for *Phytophthora* species from Miles et al., 2015. Isothermal assay primers TrnM-F and TrnM-R are used for genus-specific detection. ^7^ *Phytophthora* genus-specific (PhyG_ATP9_2FTail and PhyG-R6_Tail, Atp9-F) and species-specific (Psojae-nad9-R for *Phytophthora sojae* and Psan-nad9-R for *Phytophthora sansomeana*) primers from Rojas et al., 2017.

Non-isothermal molecular-based assays (e.g., polymerase chain reaction (PCR) and quantitative PCR (qPCR)) have been developed to identify *Phytophthora* species using several nuclear and mitochondrial loci (e.g., *ypt1* gene and *atp9*–*nad9* spacer region) [1,11,12,13,14,15,16,17,18,19,20] (Figure 1). Figure 1 details the loci commonly used for *Phytophthora* species identification in both isothermal and non-isothermal assays; citations for the primers used are included in the figure legend (Figure 1). While non-isothermal molecular assays can identify *Phytophthora* species, depending on the loci amplified, molecular assays have the potential to cross react with non-target DNA [6,11]. Polymerase chain reaction-based assays require a significant amount of setup and run time, and some can cross react with certain *Pythium* or *Phytopythium* species, making them not suitable for the fast turnaround times and accuracy needed when detecting and identifying plant pathogens [1]. Likewise, PCR assays require gel electrophoresis of the DNA product and cannot be performed in the field for onsite detection. qPCR assays that do not need gel electrophoresis for results, instead using a probe containing a fluorophore to detect amplification on a fluorometer with the ability to quantify the target DNA, are available. However, significant time input is still needed to perform DNA extractions and run the qPCR assay itself.

Isothermal assays used in plant diagnostics are predominately loop-mediated isothermal amplifications (LAMPs) or recombinase polymerase amplifications (RPA) [19,20,21,22,23,24,25,26,27,28,29,30,31,32]. These assays achieve amplification at stable temperatures (65 °C for LAMP and 39–42 °C for RPA assays) and thus do not need thermocyclers to amplify target DNA. As with any molecular-based assay, there can be issues with cross contamination in a laboratory setting. However, isothermal assays such as LAMP and RPA have the potential to be a fast and reliable test to determine the presence or absence of an organism within a sample [31].

The majority of the currently available isothermal diagnostic assays for *Phytophthora* are LAMP assays. Currently, LAMP assays have been reported for *Phytophthora kernoviae* [21], *Phytophthora infestans* [22], *Phytophthora cinnamomi* [23], *Phytophthora melonis* [24], *Phytophthora nicotianae* [25], *Phytophthora sojae*, and *Phytophthora ramorum* [21,26]. LAMP assays utilize four primers designed to anneal to different regions of the target DNA, as well as a unique DNA polymerase, with strand displacement activity enabling target amplification at a constant temperature (65 °C) [33]. Monitoring to determine a successful amplification can be performed visually, as a magnesium pyrophosphate precipitate is produced as the assay runs or fluorescent dyes, such as SYBR Green, can be incorporated so that the assay can be detected on a fluorometer [34]. The primary disadvantage is that the chemistry is quite different from PCR and so it may take significant optimization to achieve successful and specific amplification [35]. Likewise, the large number of amplicons produced in LAMP reactions make them difficult to use in the lab without amplicon contamination occurring. Limited information is available about multi-plexing LAMP reactions or whether it is possible to use this technology for detection of specific SNPs.

RPA isothermal assays have been developed for many plant pathogens [19,20,27,28,29,30,31,32]. For *Phytophthora* species specifically, a genus-specific assay (targeting the *trnM*-*trnP*-*trnM* gene order) and four species-specific assays targeting the *atp9*–*nad9* spacer region have been validated as specific [19,20]. RPA assays are more specific than using antibody based immunostrips and enzyme-linked immunosorbent assays (ELISA), as they rely on conserved DNA sequences instead of a generic antigen for detection [10,36]. RPA assays, like LAMP, are isothermal, with amplification typically occurring between 39 and 42 °C, and thus do not need a thermocycler to amplify the target region of DNA. Due to the simplicity in hardware and reagents required, these RPA assays are suitable for field deployment and can be used to provide real-time identification of targeted microbes within a single sample in under 30 min with minimal equipment inputs [19,20], making them a suitable replacement for current serological techniques [37]. Unlike LAMP assays, commercially available RPA exo kits (TwistAmp^®^ exo, TwistDX Ltd., Cambridge, UK) have a DNase to digest the amplified template, thus reducing the probability of amplicon contamination.

Commercially available RPA kits with either lyophilized or liquid reagents are available through limited sources (TwistDX Ltd., Cambridge, UK and Agdia, Elkhart, IN, USA). The lyophilized formulation only requires a rehydration buffer, magnesium acetate, user-supplied primers, probes and DNA template to start the amplification. However, there are currently no preformulated kits with primers and probes incorporated into the lyophilized pellet commercially available for oomycete pathogen detection. Having preformulated kits containing primers and probes from previously validated *Phytophthora* genus detection assays [20] would facilitate use by regulatory agencies and diagnostic laboratories working with *Phytophthora* species, as it would simplify their use under field conditions. In this manuscript, a previously developed assay that was validated against more than 136 *Phytophthora*, 21 *Pythium* and 1 *Phytopythium* species to assure its specificity to only amplify *Phytophthora* sp. was used to determine whether lyophilization negatively affects the sensitivity and specificity of the assay. The goal of this study was to (1) evaluate whether lyophilization of *Phytophthora* genus-specific primers and probes within RPA assay tubes affects the sensitivity and specificity of the assay and (2) evaluate whether the preformulated lyophilized *Phytophthora* genus-specific RPA assay is transferable between multiple qPCR platforms and labs using a single-blind DNA sample testing method.

## 2. Results

### 2.1. Comparable Results Were Observed between Preformulated and Commercially Available RPA Reactions

**Figure 2:**
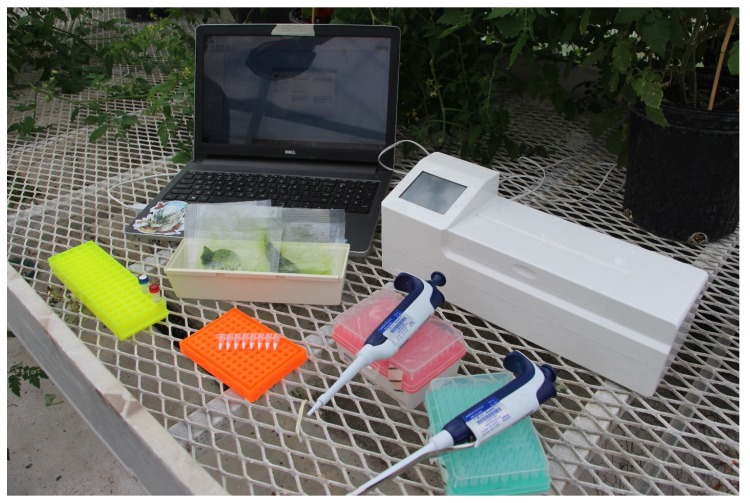
Visualization of the minimal space and equipment needed to perform the preformulated recombinase polymerase amplification (RPA) assay outside of the laboratory. AxxinT16-ISO used for visualization.

**Figure 3:**
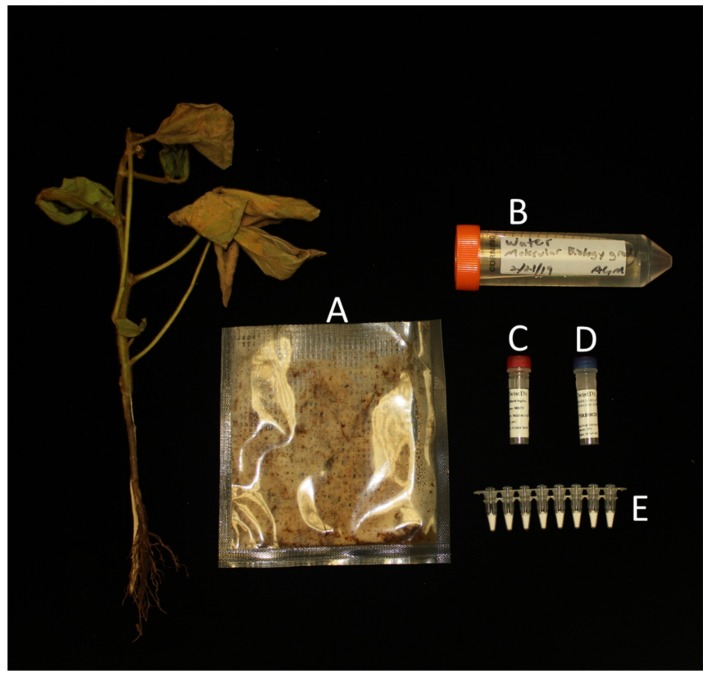
All necessary reagents to run the lyophilized RPA assay. (**A**) Crude extract produced with diseased plant sample and GEB2 buffer within an Agdia mesh bag. (**B**) Sterile water. (**C**) Magnesium acetate to initiate the reaction. (**D**) Rehydration buffer. (**E**) Preformulated lyophilized RPA reagents in pre-loaded tubes (TwistDX, Cambridge, UK).

To test the preformulation of the RPA assay, the authors collaborated with TwistDx to manufacture a preliminary batch of the preformulated assay using the TwistDx TwistAmp exo kits. These kits contained primers and probes lyophilized with nearly all other reagents, requiring relatively few reagent additions and minimal equipment (Figure 2 and Figure 3). This preliminary batch of the preformulated assay was used to identify whether there was an effect of preformulation on assay sensitivity or specificity. Initially, the preformulated assay was validated using the Axxin T16 to identify whether the limit of detection (LOD) of the assay would be comparable to the commercially available assay. Overall, the preformulated assay performed very similar to the commercially available assay, with an average onset of amplification time difference of 43.8 s between the preformulated and commercially available kits, a negligible difference in assays that run for 30 min (Table 1). The preformulated assay was able to identify *P. ramorum* in crude infected samples, as well as purified *P. cinnamomi* DNA. The plant internal control was detected in all samples containing plant tissue but was not detected when using purified DNA extractions from cultured oomycetes only (Table 1).

### 2.2. Multiple Platforms Were Effective at Detecting Phytophthora Species by RPA

The Axxin T16-ISO isothermal (Martin lab), BioRad CFX96 (Chilvers lab), QuantStudio 6 (Blomquist lab) and Applied Biosystems ViiA7 (Bilodeau lab) platforms were used to further evaluate the transferability between labs of the preformulated *Phytophthora* RPA assay using *Phytophthora ramorum*-purified DNA, tested at various concentrations ranging from 0.33 to 0.33 pg/µL, with and without a crude plant extract (Table 2). Crude plant extracts were added to the purified DNA to verify assay specificity and to simulate reactions with actual plant samples at various pathogen DNA concentrations. A positive control (500 pg/µL purified *P. ramorum* DNA) and negative control (500 pg/µL purified *Pythium splendens* DNA) were also tested in each experiment. The limit of detection (LOD) for purified DNA without plant extract was 33 pg/µL for the CFX96 and ViiA7 qPCR platforms (Table 2). The addition of a crude plant extract to the purified DNA had no effect on LOD for the ViiA7 qPCR platform, while the CFX96 could only detect down to 0.33 ng/µL in the presence of a crude plant extract (Table 2). Py. splendens DNA did not amplify in all four platforms. The Axxin T16-ISO platform had a single instance of a false-positive reading for the plant internal control (Table 2). The Axxin T16-ISO and QuantStudio 6 were slightly more sensitive and able to detect *P. ramorum* consistently at 3.3 pg of DNA, with and without plant extracts added.

### 2.3. Accurate Identification of Phytophthora-Infected Material Was Possible in a Single Blind Sample Evaluation of the Preformulated Assay

Samples collected during a 2015 *Phytophthora* survey of California nurseries [20] were used as a single-blind validation panel for further assay validation. The causal agent of the diseased tissues was identified with high confidence using isolation, qPCR and commercial RPA methods previously [20]. A blind validation panel was constructed by taking a subset of these samples and removing the identities before being sent to all labs participating in the evaluation. Results from Miles et al. [20] (Axxin T16-ISO) were then treated as a standard for pathogen identification when testing the preformulated *Phytophthora* genus-specific RPA assay. The BioRad CFX96 platform was able to accurately replicate the Axxin T16’s *Phytophthora* genus qPCR and commercial RPA results on both *Phytophthora*-infected and non-infected plant tissues (Table 3). The Applied Biosystems ViiA7 had a single technical rep with a false-negative reading with the plant internal control. However, all *Phytophthora*-infected samples tested with the ViiA7 platform had a positive amplification, while all *Phytophthora* free samples did not amplify (Table 3). The QuantStudio 6 platform had a single instance of false-positive readings for the *Phytophthora* genus-specific assay: *Asparagus officianalis* infected with *Pythium* sp. Likewise, this platform also had three instances of false-negative readings for amplification with the *Phytophthora* genus-specific assay. There was only one instance of a false-negative reading for the plant internal control (Table 3).

### 2.4. Phytophthora-Infected Fresh Plant Material Can Be Identified in under 15 Min

Leaves of a *Rhododendron* plant were inoculated individually with five *Phytophthora* species and a mock-inoculation control. All *Phytophthora*-infected leaves exhibited symptoms 10 dpi and were used to make crude plant extracts. Mock-inoculated leaves did not develop symptoms and were also harvested 10 dpi. The lyophilized assay was able to accurately identify all *Phytophthora*-infected leaves when using a crude plant extract, with no false-positive readings for the mock-inoculated or water-only controls (Table 4). In one amplification for the sample inoculated with *Phytophthora boehmeriae*, the plant internal control amplification was no different than the water only control (Table 4).

Soybeans used in an oomycete soil baiting assay resulted in soybean seedlings displaying symptoms of *Phytophthora sojae* infection. Diseased seedlings were cut in half vertically, where half was used to culture the causal agent and half was used to produce crude plant extracts. *P. sojae* was successfully isolated from all seedlings, and 100% of these seedlings also resulted in a positive *Phytophthora* amplification (Table 4).

Environmental samples suspected of having a *Phytophthora ramorum* infection were submitted to the California Department of Food and Agriculture Plant and Pest Diagnostics (CDFA-PPD) lab for further testing. Causal organisms of disease were identified as described below. All *Phytophthora ramorum*-infected samples resulted in a positive amplification. One sample, “*Phytophthora chlamydospora*” had no amplification on either the *Phytophthora* genus-specific or the plant internal control. Likewise, there was one more instance of a false-negative reading with the plant internal control (Table 5). Samples were tested on the QuantStudio 6 platform.

## 3. Discussion

During evaluations of the preformulated RPA kits, to determine the effect of lyophilization on primers and probes, nearly no difference between the preformulated and commercially available kits was found. An average onset of amplification time difference of 43.8 s when using the Axxin T16-ISO RPA chamber was observed between the preformulated and commercially available kits (Table 1). Nearly indistinguishable results indicated that preformulation via lyophilization of primers and probes did not significantly affect the sensitivity and specificity of the *Phytophthora* genus-specific assay. This assay was found to be specific to *Phytophthora*, with the ability to consistently detect and differentiate between *Phytophthora*-infected and non-infected tissue. Likewise, the limit of detection (LOD) was identified using four separate platforms. The LOD using purified DNA was 33 pg with the BioRad CFX96 and ViiA7 platforms using the preformulated kits, while the Axxin T16-ISO RPA chamber and QuantStudio 6 could detect down to 3.3 pg with and without a crude plant extract. The LOD using a crude plant extract for the BioRad CFX96 was 330 pg, whereas the LOD for the ViiA7 system was 33 pg.

There was an observed difference in sensitivity of the assay when using the Axxin T16-ISO compared to the qPCR platforms, with the Axxin being 10–100-fold more sensitive. This is likely due to the Axxin FAM fluorophore channel being scaled to a high level of sensitivity prior to testing. The Axxin T16-ISO platform had a single instance of a false-positive reading with the plant internal control. Similarly, when testing fresh samples of soybean, *Rhododendron* and environmental samples, differences in detection of *Phytophthora* and the plant internal control were identified. The BioRad CFX96 had consistent amplification of both the plant internal control as well as positive *Phytophthora* amplification in *Phytophthora sojae*-infected soybeans. However, the ViiA7 RT-PCR system and QuantStudio 6 had instances of inconsistent amplification of the plant internal control and *Phytophthora*-infected plants, where one of the two technical reps did not amplify when both plant material and *Phytophthora* were present, or in the case of the QuantStudio 6, no amplification was detected. This could be due to amplification starting in some reactions before the samples were placed into the respective platforms. Inadequate mixing of the crude plant extract and reagents within tubes could cause variable amplification efficiency between technical replications. Varying levels of plant DNA within a plant extract could also lead to variable amplification times of the plant internal control. In general, reaction times under 30 min would be considered positive for both the *Phytophthora* genus-specific marker and the plant internal control. Despite the observed instances of variability in amplification, approximately 90% of reactions were identical across the three tested qPCR platforms as compared to the commercially available RPA kit as well as the *Phytophthora* genus-specific qPCR assay run on the Axxin T16-ISO (Table 3). These results reinforce our hypothesis that preformulation within a single pellet will not negatively affect assay performance.

When used in a plant inspection setting, the preformulated RPA was able to accurately identify all *Phytophthora ramorum*-infected samples (Table 5). Likewise, closely related genera like *Plasmopara* and *Pythium* were not amplified—another example of the specificity of this assay. Interestingly, one *Phytophthora* sample, *Phytophthora chlamydospora*, did not amplify. This could be due to high levels of inhibitors being present in the lysate, as the plant internal control also did not amplify. Nevertheless, in a diagnostic setting, when interpreting a molecular diagnostic test, it is imperative to take into account the history of the sample to accurately identify the causal organism. Samples displaying symptoms typical of a *Phytophthora* disease (i.e., water soaked, necrotic stem vascular tissue in soybeans indicative of *P. sojae*, necrosis on the margins of *Rhododendron* leaves indicative of *P. ramorum*) which have ambiguous results from molecular tests should be more thoroughly investigated via microscopy, culturing techniques or DNA sequencing to identify the causal organism.

The ability to test and diagnose quarantined plant pathogens quickly is imperative to stop the spread of these destructive plant diseases. Furthermore, the ability to conduct in field testing for these pathogens could reduce the time between diseased plant observation and causal agent identification. Isothermal assays, such as RPA, are useful in this regard, as they can be field deployable when using an isothermal detection chamber, such as the Axxin T16-ISO (Figure 2). Likewise, the assay validated in this manuscript requires minimal reagents to be added, as the primers and probes are preformulated into the lyophilized pellet (Figure 3). While the Axxin T16-ISO was used in this study, there are similar fluorimeters available from various manufacturers that could also be used: BioRanger (Diagenetix Inc., Honolulu, HI, USA), Amplifire (Agdia Inc., Elkhart, IN, USA), Genie^®^ II or Genie^®^ III (OptiGene Ltd., West Sussex, UK).

Commercial availability of an accurate, field-deployable, isothermal assay preformulated with necessary primers and probes that does not require expensive lab equipment, DNA extraction or constant refrigeration of reagents would benefit in-field diagnostics. Wide availability of such an assay could decrease the time between observation of disease and identification of the causal agent of disease, something that would be of particular importance for regulated *Phytophthora* species like *Phytophthora ramorum* and *P. kernoviae*. Having an assay that can detect regulated and non-regulated pathogens increases its potential use from diagnostics labs working with regulated *Phytophthora* species to labs that receive potentially infected plant samples from the horticultural and agricultural industries for identification, as well as labs conducting surveys on *Phytophthora* species. The lyophilized isothermal RPA assay developed and validated in this manuscript could be used for any such aforementioned study on *Phytophthora* species.

The isothermal RPA assay described in this manuscript uses mitochondrial gene order to provide sensitivity and specificity to *Phytophthora* species. The mitochondrial gene order, *trnM-trnP-trnM*, is conserved among *Phytophthora* species, making it highly specific [19,20], unlike other regions previously used for diagnostic assays [1,11,12,13,14,15,16,17,18,19,20,38]. Interestingly, this region is not conserved in the closely related genera *Pythium*, making it an ideal marker for *Phytophthora*-specific diagnostics. This gene region has been previously developed and validated into other TaqMan and RPA *Phytophthora* genus- and species-specific assays [19,20,39,40]. Miles et al. [20] developed a *Phytophthora* genus-specific isothermal assay with a detection limit between 200 and 300 femtograms (0.2–0.3 pg). The TaqMan assay for *Phytophthora* genus developed by Rojas et al. [19] had a limit of detection of 100 fg (0.1 pg) when using purified DNA, while the RPA assay developed had a limit of detection of 10 pg [19]. The preformulated lyophilized assay evaluated herein had nearly identical results to that of the standard commercially prepared kit; it was able to detect purified *Phytophthora* species DNA down to 3.3–33 pg depending on which platform was used to read amplification (Table 1 and Table 2) [20].

In addition to the *trnM-trnP-trnM* gene order, another gene order (atp9–nad9) was also found to be useful for development of species-specific detection capabilities. Bilodeau et al. [40] and Miles et al. [39] also developed fifty species-specific *Phytophthora* TaqMan probes based on the *atp9–nad9* spacer region for regulated and non-regulated *Phytophthora* species, as well as one-hundred and twenty-four unique TaqMan probes for various *Phytophthora* species. RPA assays targeting this locus were developed for detection of *Phytophthora* of regulatory importance (such as *Phytophthora ramorum* and *P. kernoviae*) or of agronomic importance (*Phytophthora sojae*, *P. cactorum*, *P. citrophthora*) and could be used with the *trnM-trnP-trnM* assay to provide further, species-specific, diagnostic capabilities [20,39,40].

The potential for false-negative readings and false-positive readings has been a long-standing issue in plant pathogen serological and molecular diagnostics [38,41]. In order to control for false-positive readings, samples should always be run in two to three technical reps, so that false-positive readings for amplification can be identified by consensus. Likewise, including a water control reaction can identify potential contamination of reagents, which is of concern with molecular assays and can lead to false-positive readings for amplification. Unlike LAMP assays that generate DNA end products that could contaminate pipettes or the workspace leading to future false-positive readings, the RPA exo formulation used in this experimentation has an exonuclease component to the amplification mixture that digests the DNA during amplification, thereby reducing the potential for cross contamination. To control for false-negative readings due to the presence of amplification inhibitors, our assay utilizes a plant mitochondrial internal control primer and probe set which targets the plant cytochrome oxidase (*cox*) gene [20]. This was used to determine whether inhibitors in the plant extract prevented amplification, which provides a control amplification for quality of the extract. Providing an internal control for each reaction allows users to quickly identify whether reactions needs to be repeated. Assays performed with purified DNA from *Phytophthora* species could enable a similar internal control by ‘spiking’ their reactions with pathogen-free plant DNA.

One interesting aspect of the isothermal RPA assay is the potential use of a reverse-transcriptase (RT) to target RNA without the need to construct cDNA before use [42]. Previous studies have shown that this can be done with viral plant pathogens such as plum pox virus of *Prunus* sp. [30], little cherry virus 2 [29], as well as Potato virus Y and Wheat dwarf virus [42]. Using RT in an RPA assay could provide an in-field diagnostic test to identify living *Phytophthora* pathogens within samples by targeting mRNA for a constitutively expressed gene [43,44]. Real-time identification of a living pathogen-causing disease would be of great benefit to plant pathology as a whole, as the main criticism of current molecular diagnostic procedures is that there is no way to identify whether the DNA that was amplified was from viable or dead cells. Having the ability to amplify RNA templates by RPA would solve this problem, as RNA is not persistent in the environment like DNA is, thus ensuring that the amplification is from a viable cell. Other procedures to ensure that only DNA from living cells is amplified have been developed using propidium monoazide, a membrane-impermeant dye that intercalates into DNA in the absence of an intact membrane, inhibiting amplification [45]. However, more testing needs to be done in this area before it can be widely adopted for practical use in molecular diagnostics.

In conclusion, given the consistency of results obtained by different labs using different equipment, the preformulated assay described here would be a useful tool to plant diagnostic labs and points of inspection if commercially available. Specifically, this assay could be used during routine inspections at nurseries and ports of entry to screen materials for regulated *Phytophthora* species, such as *Phytophthora ramorum* and *P. kernoviae*. Samples with *Phytophthora*-positive amplification could be identified in under 30 min without the need to refrigerate the reagents or use a non-portable thermocycler, giving inspectors the ability to perform these reactions on site and in real time. This assay is sensitive (LOD of 3.3–33 pg DNA) and specific to *Phytophthora* species, does not cross react with *Pythium* or closely related organisms [20], and does not require extensive DNA preparations before use. Based on the ease of use, minimal equipment needed, sensitivity and specificity, the assay described in this manuscript would be of benefit and use to plant diagnostic labs and inspectors at ports of entry to monitor regulated or non-regulated *Phytophthora* species.

## 4. Materials and Methods

### 4.1. Reagents and Assay Conditions

Preformulated lyophilized RPA assay tubes in the TwistAmp exo formulation containing (1) the *Phytophthora* genus-specific primers and probes for the *trnM-trnP-trnM* locus and (2) the primers and probes for the plant internal control, targeting the plant *COX1* gene, of Miles et al. (2015) were prepared by TwistDX (Cambridge, UK) (Table 6). Concentrations were as previously reported. The pellet was rehydrated and completely dissolved with 37.5 μL TwistAmp exo rehydration buffer and 7 μL DNase-free water prior to adding 3 μL of crude plant extract or purified DNA obtained as described below. This mixture was homogenized via pipette and 2.5 μL of 280 mM magnesium acetate (MgAc) was pipetted into the cap of each reaction tube and closed gently so that the MgAc remained in the cap. The samples were centrifuged for 10 s to initiate a uniform amplification starting time, mixed, and incubated at room temperature for 4 min, mixed again, centrifuged again, and placed into the Axiin T16 and qPCR platforms (BioRad CFX96, Bio-Rad Laboratories, Hercules, CA, USA, QuantStudio 6, Thermo Fisher Scientific Inc., Waltham, MA, USA and the ViiA7 Real-Time PCR system, Thermo Fisher Scientific Inc.). FAM (*Phytophthora* genus probe) and ROX (plant internal control probe) channels were used on each qPCR platform to measure fluorescence over a 30 min period at 39 °C. The fluorescence cycle threshold (CT) baseline for FAM and ROX was set just above the water control for each platform, so that samples with equal or less fluorescence than the water control were identified as negative. Data was exported into Microsoft^®^ Excel (2007) to produce tables.

### 4.2. Production of Pure DNA and Crude Plant Extracts

Pure DNA samples used in this study were obtained via a modified phenol-chloroform extraction protocol [46]. DNA samples were quantified using the Quant-iT^™^ dsDNA High Sensitivity assay on the Invitrogen^™^ Qubit^™^ 2 system (Thermo Fisher Scientific). Serial dilutions of *Phytophthora ramorum* and *P. cinamommi* were made to determine the LOD of the assay, as explained below.

Crude plant extracts were made from healthy *Umbellularia californica* leaves. Briefly, 0.5 g of leaf tissue was added to 5 mL to GEB2 buffer within a netted bag and ground. A volume of 1 µL of this crude plant extract was added to purified DNA to determine the LOD of the assay with and without a crude plant extract, as described below.

### 4.3. Initial Evaluation of Preformulated lyophilized Kits Reaction

A preliminary experiment was conducted to identify the effect, if any, that primer/probe lyophilization had on the RPA assay. Purified *Phytophthora cinnamomi* DNA [20], ranging from 0.35 to 3500 pg, was used to compare the reaction containing lyophilized primers/probe to the commercially available formulation. Crude plant *Phytophthora ramorum*-infected extracts were used to validate that the assay still worked when lyophilized, and purified *Pythium splendens* DNA was used as a negative control (Table 1). The Axxin T16 platform was used for data collection.

### 4.4. Limit of Detection Determination for Preformulated Lyophilized Kits

Once the assay with lyophilized primers/probe was observed to work as expected, the BioRad CFX96, Axxin T16, QuantStudio 6 and the ViiA7 Real-Time PCR system were used for further evaluation of the preformulated reactions. Purified DNA from *Phytophthora ramorum* was used as a positive control (500 pg/µL) and purified *Pythium splendens* DNA was used as a negative control (500 pg/µL) in both systems [20,39]. Purified DNA from *P. ramorum* was further used to determine the LOD of the platforms using serial dilutions ranging from 0.33 to 0.33 pg/µL. The assay LOD was determined with and without plant extract for each qPCR platform (Table 2).

### 4.5. Single Blind Multi-Lab Evaluation of Preformulated Lyophilized Kits Reaction

Plant samples used for the single blind lyophilized RPA evaluations were obtained during a 2015 survey of California nurseries for *Phytophthora* diseases [20]. Putative *Phytophthora*-infected samples were diagnosed and identified via culturing, qPCR, and RPA to identify the causal organism of infection. A subset of samples from this survey were randomly selected to be used in the preformulated RPA evaluation. Single blind testing was performed on *Phytophthora*- and *Pythium*-infected plant samples and compared to commercial RPA and qPCR reactions using the Axxin T16, as well as direct isolations from the infected material for identification (Table 3). The BioRad CFX96, QuantStudio 6 and ViiA7 Real-Time PCR platforms were used for data collection.

### 4.6. Fresh Sample Testing

Fresh plant samples from soybean seedlings and *Rhododendron* leaves were used to further test the assay on the BioRad CFX96 and ViiA7 platforms, respectively (Table 4). Soybean seedlings were used from a soil baiting assay for *Phytophthora sojae* [47]. Diseased seedlings were identified and carefully removed from soil. Seedlings were then washed of any debris and surface sterilized in a 70% ethanol solution before being cut in half vertically, with half of a single seedling used as a crude extract for RPA assays and the other half used for isolation and pathogen identification. Isolations were conducted on corn meal agar medium (CMA-PARP) amended with, pentachloronitrobenzene (PCNB) (50 mg/L), ampicillin (250 mg/L), rifampicin (10 mg/L and pimaricin (5 mg/L), selecting for oomycetes [48]. *Phytophthora sojae* was isolated from all soybean seedlings and identified based on host, culture morphology on CMA-PARP media as well as its inability to grow on full-strength PDA [47]. Crude soybean extracts were prepared by adding 2.5 g plant tissue into 5 mL GEB2 buffer (product number: ACC 00130; Agdia) in plastic extraction bags containing netting (product number ACC 00930; Agdia).

Detached *Rhododendron* leaves were inoculated individually with *Phytophthora boehmeriae*, *P. pseudosyringae*, *P. cryptogea*, *P. ramorum*, *P. infestans*, or a mock-inoculated leaf and then tested with the lyophilized assay on the ViiA7 qPCR platform (Table 4). Leaves were surface sterilized with a 10% bleach solution for 1 min and subsequently washed twice with sterile water before inoculation. Inoculations were performed by injuring the leaf using a needle and placing a colonized agar plug of the pathogen onto the injury site, or a non-colonized agar plug for the mock inoculation. All leaves inoculated with *Phytophthora* species exhibited symptoms, while the mock-inoculated leaf did not develop symptoms. Inoculated leaves were harvested 10 days post inoculation (DPI) and a crude plant extract was made as noted above.

Environmental samples submitted to the California USDA-ARS for Sudden Oak Death (*Phytophthora ramorum*) testing were used to validate this assay in a plant inspection setting. Samples submitted were ground in Lysing Matrix A (product number: SKU 6910100, MP Biomedicals) following the Agdia *Phytophthora* ELISA Protocol (product number: PSA 92601; Agdia). The samples were then centrifuged to pellet the solid organic material and the lysate was used for RPA testing on the QuantStudio 6 platform. Causal organisms of disease were verified via ELISA and ITS sequencing (Table 5).

## Figures and Tables

**Table 1:** Comparison of recombinase polymerase amplification (RPA) assays using commercially available kits requiring addition of primers and probes and preformulated kits with the primers and probes lyophilized with the reaction mixture. Recombinase polymerase amplification using primers and probes for *Phytophthora* genus-specific detection and a plant internal control reported by Miles et al. [20]. Data collected with an Axxin T16-ISO platform.

Sample	Average Time Onset of Amplification with the Commercially Available Reaction ^a^	Average Time Onset of Amplification with the Preformulated Lyophilized Reaction ^a^
Crude plant extract *Phytophthora rubi*-infected raspberry cane	**17.4** ^b^	**17.6**
	*8.0* ^b^	*8.0*
Crude plant extract infected *Phytophthora ramorum*-leaf	**15.7**	**15.2**
	*8.1*	*8.1*
Crude plant extract infected *P. ramorum*-leaf	**14.8**	**12.3**
	*8.0*	*8.0*
Purified *Pythium splendens* DNA (1 ng)	- ^c^	-
	-	-
Purified *Phytophthora cinnamomi* DNA (3500 pg)	**6.5**	**5.85**
Purified *P. cinnamomi* DNA (350 pg)	**9.0**	**8.5**
Purified *P. cinnamomi* DNA (35 pg)	**11.4**	**11.0**
Purified *P. cinnamomi* DNA (3.5 pg)	**16.5**	**16.1**
Purified *P. cinnamomi* DNA (0.35 pg)	-	-

^a^ Values are expressed as the average (n = 2) onset of amplification in minutes, including the pre-agitation step. ^b^ Bold (top) values are *Phytophthora* (FAM). Italic (bottom) values are plant internal control (ROX). ^c^ (-) indicates a reaction no different than the water control.

**Table 2:** Initial validation of preformulated lyophilized RPA assay on crude plant extracts and purified *Phytophthora ramorum* or *Pythium splendens* DNA.

Sample	Platform	Average Time Onset of Amplification without Plant Extract ^a^		Average Time Onset of Amplification with Plant Extract	
		*Phytophthora* Genus (FAM) ^b^	Plant Internal Control (ROX) ^c^	*Phytophthora* Genus (FAM)	Plant Internal Control (ROX)
*P. ramorum* (500 pg/µL) (Positive control)	Axxin T16-ISO	7.66	NR ^d^	- ^e^	-
	Bio-Rad CFX96	12.38	NR	-	-
	ViiA7 RT-PCR	13.01	NR	-	-
	QuantStudio 6	6.69	NR	-	-
*Phytophthora*-infected citrus	Axxin T16-ISO	-	-	7.24	8.21
	Bio-Rad CFX96	-	-	9.08	10.46
	ViiA7 RT-PCR	-	-	12.68	14.35
	QuantStudio 6	-	-	5.55	5.34
*Pythium splendens* (500 pg/µL) (Negative Control)	Axxin T16-ISO	NR	NR	-	-
	Bio-Rad CFX96	NR	NR	-	-
	ViiA7 RT-PCR	NR	NR	-	-
	QuantStudio 6	NR	NR	-	-
*P. ramorum* (0.33 ng/µL)	Axxin T16-ISO	10.54	12.67	12.11	10.04
	Bio-Rad CFX96	14.97	NR	17.36	29.81
	ViiA7 RT-PCR	12.6	NR	13.99	8.95
	QuantStudio 6	7.53	NR	8.11	23.21
*P. ramorum* (33 pg/µL)	Axxin T16-ISO	18.90	NR	19.71	14.22
	Bio-Rad CFX96	22.66	NR	NR	20.45
	ViiA7 RT-PCR	16.51	NR	16.98	9.68
	QuantStudio 6	9.31	NR	11.83	22.77
*P. ramorum* (3.3 pg/µL)	Axxin T16-ISO	27.32	NR	31.12	13.77
	Bio-Rad CFX96	NR	NR	NR	10.57
	ViiA7 RT-PCR	NR	NR	NR	9.57
	QuantStudio 6	12.99	NR	16.01	23.09
*P. ramorum* (0.33 pg/uL)	Axxin T16-ISO	NR	NR	NR	10.06
	Bio-Rad CFX96	NR	NR	NR	15.46
	ViiA7 RT-PCR	NR	NR	NR	9.28
	QuantStudio 6	NR	NR	NR	22.97

^a^ Values are expressed as the average (n = 2) onset of amplification in minutes. ^b^ *Phytophthora* genus-specific probe. ^c^ Plant internal control probe. ^d^ (NR) refers to reactions that were run but did not have any observed amplification. ^e^ (-) refers to reactions that could not be run with or without a plant extract, respectively.

**Table 3:** Blind validation panel used to evaluate the preformulated lyophilized recombinant polymerase amplification (RPA) reaction and commercially available RPA reactions compared to the results of qPCR assays as reported in Miles et al. [20].

		Axxin T16 ^a^		Bio-Rad CFX96 RPA		ViiA7 System RPA			QuantStudio 6 RPA	
Sample	Pathogen Identified	*Phytophthora* Genus RPA (FAM) ^b^	*Phytophthora* Genus qPCR (FAM)	*Phytophthora* Genus (FAM)	Plant Internal Control (ROX) ^c^	*Phytophthora* Genus (FAM)	Plant Internal Control (ROX)	*Phytophthora* Genus (FAM)		**Plant Internal Contro** **(ROX)**
*Rhamnus californica*	*Phytophthora cactorum*	+ ^d^	+	+	+	+	+	+		+
*Prunus avium*	*Pythium* sp.	− ^e^	−	−	+	−	+	−		−
*Gardenia jasminoides ‘Radicans’*	*P. nicotianae*	+	+	+	+	+	+	−		+
*Gardenia jasminoides ‘Mystery’*	*P. nicotianae*	+	+	+	+	+	−	+		+
*Aucuba japonica ‘Mr. Goldstrike’*	*P. citricola*	+	+	+	+	+	+	+		+
*Asparagus officinalis*	*Pythium* sp.	−	−	−	+	−	+	+		+
*Rhus integrifolia*	*P. nicotianae*	+	+	+	+	+	+	+		+
*Fragaria x ananassa*	*P. cactorum*	+	+	+	+	+	+	−		+
*Rubus* sp.	*Pythium* sp.	−	−	−	+	−	+	−		+
*Rubus* sp.	*P. rubi*	+	+	+	+	+	+	+		+
*Citrus* sp. 1	*P. citrophthora*	+	+	+	+	+	+	+		+
*Citrus* sp. 2	*P. citrophthora*	+	+	+	+	+	+	+		+
*Citrus* sp. 3	*P. citrophthora*	+	+	+	+	+	+	+		+
*Citrus* sp. 4	*P. citrophthora*	+	+	+	+	+	+	+		+
*Citrus* sp. 5	*P. citrophthora*	+	+	+	+	+	+	+		+
*Myrtus* sp.	*P. nicotianae*	+	+	+	+	+	+	+		+
*Pseudotsuga menziesii*	*P. cambivora*	+	+	+	+	+	+	+		+
*Hedera* sp.	*P. tropicalis*	+	+	+	+	+	+	+		+
*Rhododendron* sp.	*P. ramorum*	*+*	*+*	+	+	+	+	+		+
*Rhododendron* sp.	*P. ramorum*	*+*	*+*	+	+	+	+	−		+
*Umbellularia californica*	*P. ramorum*	*+*	*+*	+	+	+	+	+		+
*Viburnum* sp.	*P. ramorum*	*+*	*+*	+	+	+	+	+		+
*Rhododendron* sp.	*P. ramorum*	*+*	*+*	+	+	+	+	+		+
Water	N/A	−	−	−	−	−	−	−		−

^a^ Data collected by Miles et al. 2015. ^b^ *Phytophthora* genus-specific probe. ^c^ Plant internal control probe. ^d^ (+) indicates a positive amplification. ^e^ (−) indicates a negative amplification, no different than the water control.

**Table 4:** Horticulture (*Rhododendron* sp.) and agriculture (soybean) samples tested with the preformulated lyophilized recombinase polymerase amplification (RPA) assay.

Phytophthora Species ^a^	Host Plant	Platform	Phytophthora Genus (FAM)	Plant Internal Control (ROX)
			Mean OT ^b^	Mean OT
*P. boehmeriae*	*Rhododendron* sp.	ViiA7 RT-PCR	6.78 *	NR
*P. pseudosyringae*	*Rhododendron* sp.	ViiA7 RT-PCR	10.63	5.92 *
*P. cryptogea*	*Rhododendron* sp.	ViiA7 RT-PCR	9.52	6.96
*P. ramorum*	*Rhododendron* sp.	ViiA7 RT-PCR	9.7	7.41
*P. infestans*	*Rhododendron* sp.	ViiA7 RT-PCR	6.20	10.74 *
*P. infestans*	*Rhododendron* sp.	ViiA7 RT-PCR	8.01	9.84 *
Leaf only	*Rhododendron* sp.	ViiA7 RT-PCR	NR ^c^	8.15 *
Water only	*Rhododendron* sp.	ViiA7 RT-PCR	NR *	NR *
*P. sojae* 1	*Glycine max*	Bio-Rad CFX96	8.82	11.76
*P. sojae* 2	*Glycine max*	Bio-Rad CFX96	6.88	8.04
*P. sojae* 3	*Glycine max*	Bio-Rad CFX96	5.36	7.46
*P. sojae* 4	*Glycine max*	Bio-Rad CFX96	4.35	7.92
*P. sojae* 5	*Glycine max*	Bio-Rad CFX96	3.97	10.8
*P. sojae* 6	*Glycine max*	Bio-Rad CFX96	10.58	13.45
*P. sojae* 7	*Glycine max*	Bio-Rad CFX96	9.54	15.85
*P. sojae* 8	*Glycine max*	Bio-Rad CFX96	9.25	12.99
*P. sojae* 9	*Glycine max*	Bio-Rad CFX96	4.36	27.18
*P. sojae* 10	*Glycine max*	Bio-Rad CFX96	5.25	22.84

^a^ Species of *Phytophthora* inoculated (ViiA7) or isolated (CFX96) from host. ^b^ Values are expressed as the average (n = 2) onset of amplification (OT) in minutes. ^c^ (NR) indicates a negative reaction, no different than the water control. * n = 1.

**Table 5:** Environmental samples tested with the preformulated RPA assay on the QuantStudio 6 platform.

Sample ^a^	Host Name	*Phytophthora* Genus (FAM)	Plant Internal Control (ROX)
		Mean OT ^b^	Mean OT ^b^
*Phytophthora ramorum* 1	*Rhododendron* sp. ‘Cunningham’	3.80	6.23
*P. ramorum* 2	*Rhododendron* sp. ‘Cunningham’	7.83	26.9
*P. ramorum* 3	*Rhododendron* sp. ‘Cunningham’	5.64	5.55
*P. ramorum* 4	*Rhododendron* sp. ‘Cunningham’	5.90	5.99
*P. ramorum* 5	*Rhododendron* sp. ‘Cunningham’	7.07	6.32
*P. ramorum* 6	*Umbellularia californica*	8.39	26.9
*P. ramorum* 7	*Rhododendron* sp. ‘Grace Seabrook’	10.28	5.19
*P. ramorum* 8	*Rhododendron* sp. ‘Grace Seabrook’	8.09	5.56
*P. ramorum* 9	*Rhododendron* sp. ‘Grace Seabrook’	11.14	5.12
*P. ramorum* 10	*Rhododendron* sp. ‘Grace Seabrook’	12.78	6.12
*P. ramorum* 11	*Rhododendron* sp. ‘Grace Seabrook’	10.32	NR ^c^
*P. ramorum* 12	*Rhododendron* sp. ‘Taurus’	9.05	5.57
*P. ramorum* 13	*Arctostaphylos refugioensis*	11.19	6.93
*P. ramorum* 14	*Rhododendron* sp. ‘Rangoon’	7.75	5.27
Spumella-like flagellate	*Fragaria* sp.	NR	27.11
*Pythium debaryanum*	*Fragaria* sp.	NR	24.24
*Plasmopara viticola*	*Vitis vinifera*	NR	18.57
*P. multivora*	*Camellia sinensis*	4.80	22.06
*P. chlamydospora*	*Rosa* sp.	NR	NR
*P. brassicae*	*Brassica oleracea*	5.85	1.38
*P. syringae* 1	*Rhododendron* sp. ‘President Roosevelt’	5.22	6.07
*P. syringae* 2	*Rhododendron* sp.	4.76	5.38
*P. syringae* 3	*Rhododendron* sp.	5.55	6.31
*P. syringae* 4	*Rhododendron* sp.	5.85	5.69
*P. syringae* 5	*Rhododendron* sp.	5.54	6.04
*P. syringae* 6	*Arctostaphylos densiflora*	7.41	25.90
Unknown Isolate 1	*Chiosya ternate*	NR	26.0
Unknown Isolate 2	*Mahonia repens*	NR	9.91
Unknown Isolate 3	*Camellia sasanqua*	NR	24.24
Water control	NA	NR	NR

^a^ Species identified within the sample via ELISA and ITS sequencing. ^b^ Values are expressed as the average (n = 2) onset of amplification (OT) in minutes. ^c^ (NR) indicates a negative reaction, no different than the water control.

**Table 6:** Primers and probes used in this study for *Phytophthora* genus-specific detection and plant internal control.

Primers, Probes ^a^	Sequence (5′–3′)	Target
Primers		
*Phytophthora* genus specific		
TrnM-F	ATGTAGTTTAATGGTAGAGCGTGGGAATC	tRNA-M
TrnM-R	GAACCTACATCTTCAGATTATGAGCCTGATAAG	tRNA-M
Plant internal control		
Cox1-IPC-F	CATGCGTGGACCTGGAATGACTATGCATAGA	COX1
Cox1-IPC-R	GGTTGTATTAAAGTTTCGATCGGTTAATAACA	COX1
Probes		
*Phytophthora* genus specific		
TrnM-P	TAGAGCGTGGGAATCATAATCCTAATGTTG [FAM-dT] A [THF] G [BHQ1-dT] TCAAATCCTACCATCAT [3′-C3SPACER]	tRNA-M
Plant internal control		
Cox1-IPC-P	GGTCCGTTCTAGTGACAGCATTCCYACTTTTATTA [ROX- dT] C [THF] C [BHQ2-dT] YCCGGTACTGGC [3′-C3SPACER]	COX1

^a^ Primers and probes from Miles et al. 2015 [20].
